# The Future of Osteopathy

**DOI:** 10.7759/cureus.51636

**Published:** 2024-01-04

**Authors:** Bruno Bordoni, Allan R Escher

**Affiliations:** 1 Physical Medicine and Rehabilitation, Foundation Don Carlo Gnocchi, Milan, ITA; 2 Anesthesiology/Pain Medicine, H. Lee Moffitt Cancer Center and Research Institute, Tampa, USA

**Keywords:** palpation, fascia, quantum physics, osteopathic medicine, osteopathy

## Abstract

Every clinician has the duty to keep clinical and scientific knowledge updated, with the aim of improving clinical practice. Many homeostatic reactions of the organism are not yet well understood and framed by medicine or classical physics. Quantum physics offers new and multiple information to understand how the human body works, starting from the assumption that the macroscopic is managed by nanoscopic quantum coherence. The future of osteopathy should be to integrate the educational path with quantum physics.

## Editorial

How can a scientific discipline grow? Not by faith, because faith relies on memory, and memory can be fleeting or extremely subjective. Not by inertia because, when the force of the push ceases, the inertia ends. Not out of habit, because by moving to a new house, one's needs and conveniences can change. Not by imposition, because rigid science destroys itself. Osteopathy was born from the ideas of Andrew Taylor Still, DO, in the late 1800s: "Osteopathy, or osteopathic medicine, is a philosophy, a science, and an art" [1]. As a philosophy, osteopathic medicine (OM) perceives man as body and soul. As a science, OM seeks health under the aegis of clinical reasoning and research. As an art, OM finds the best salutogenic strategy for each patient. Solid matter is the expression of a quantum coherence of bosons and fermions (elementary particles) [2]. The fermion is responsible for solid matter (neutrons, protons, and electrons), while the boson represents the energy that allows the exchange of information (photons or light and phonons or sound) [2]. It is from this quantum world that physical reality arises as we perceive it with our senses. It is on these elementary particles on which human health depends and to understand constant homeostatic adaptation [3]. To give an example, the micromicidal action of neutrophils occurs with a combustion reaction, thanks to the movement of peripheral electrons (fermions); this mechanism transforms the fermion into a boson [2]. The chemical reaction (combustion or oxidoreductase) produces light or photons (bioluminescence or chemiluminescence). The elementary particles are in constant movement, to allow constant evolution. Each particle has a vibration, which is the "fingerprint" or vibrational identity. The vibration determines the characteristic of the particle itself. Particles communicate via vibration (light and sound). Communication can occur without necessarily the presence of energy (quantum tunneling) [2]. The particles can activate cellular chemical reactions without necessarily activating cell membrane receptors, by connecting directly to the deoxyribonucleic acid (DNA). The vibrations of the particles change the position of the DNA electrons, causing a reciprocal oscillatory change; on the one hand, DNA changes its functions, and, on the other hand, an indissoluble bond of mutual knowledge or quantum entanglement is created [2].

To better understand the human body's response to the osteopathic approach, the osteopathic clinician should orient his studies by also involving quantum physics. Current research is using many resources on quantum dots or semiconductor nanocrystals (nanoparticles of extremely small dimensions), relating different branches of science, exploiting the principles of quantum physics to improve the patient's therapeutic response [4]. In a recent article, Marshall highlights the concept of the importance of incorporating quantum physics to understand biology: "A growing vein of literature shows that multiple biological processes cannot be fully explained without invoking QM (quantum physics)" [5].

To give another example, in 20-30% of breast tumors, we see an increase in the expression of the human epidermal growth factor receptor 2 (HER2) receptor; this event leads to a more aggressive oncological picture. Thanks to nanomedicine and the assumptions of quantum physics, it is possible to use nanoparticles with optical and electrical properties (quantum dots), with different theranostic purposes. When quantum dots are irradiated with ultraviolet rays, these nanoparticles are activated and release biophotons; this quantum reaction allows us to better detect the quantity and site of HER2 and more specifically combat these areas with drugs.

The concepts of quantum physics also allow us to maintain our identity as clinicians with respect to the patient and, at the same time, be one. The perception of the patient's health through palpation is an action that involves many subjective sensory receptors. According to the "observer theory" of quantum physics, every time an electron connects to another electron, this bond is forever; furthermore, our intention can change the behavior of electrons, both near and distant [2]. We could state that "Every osteopathic manual evaluation is a mutual exchange of information through nanoscopic forces" and "Osteopathic touch is not just knowledge through palpation, but an act of mutual sharing" [2]. With palpation, we can influence every electron, and every electron of the patient is able to influence the perception of the clinician [2,5]. We are emitters and receivers of information. In a functional vision, these are not layers, but different levels of communication. When a tissue alters its state of rest (during palpation and before mechanotransduction), the electrons of each cell membrane affected by the perturbation move. Each movement of electrons (excitation of a cell) generates a magnetic field, while a change in potential (for excitation of the cell) or voltage generates an electric field; the result is an electromagnetic field. The movement of the electromagnetic field generates a wave (defined by mathematics and quantum physics as a soliton). When the electromagnetic wave or vibration leaves the single cell/cells, light and sound are produced. These waves will alter the electrons of nearby and distant cells, with a direction that is independent of the neural network and with a speed significantly higher than the nervous impulse. These mechanisms take place at the quantum level. We can imagine stones being thrown into the lake and the formation of many different waves, which will cross the entire lake; the whole lake is aware of the disturbance of its surface. The lake represents our body. These communication mechanisms allow the maintenance of health. When these vibrations occur in many areas and in synchronous mode, the "quantum coherence" or Larmor precession is established, which coherence allows the health status and macroscopic integrity of the tissues to be maintained [1]. Coherence facilitates the distribution of information and the continuity of the salutogenic stimulus. Electromagnetic fields millions of times weaker than the electrical voltage of the cell membrane can manipulate the metabolic behavior of the human body [1]. The osteopath's touch creates nanoscopic variations that reverberate in the macroscopic and on the health of the tissues. We are the macroscopic expression of the coherence of structures conceivable from a nanoscopic vision [1]. Osteopathy cannot be framed only in a macroscopic act but can also be conceived as a constant flow of interpenetration and knowledge at a nanoscopic level. Osteopathy is the encounter of man with man, sublimating wholeness in a gesture [1].

Science is not a dogma, but a perpetual state of change, like knowledge and technology. Science is always a journey and never an arrival. The soul of science is insecurity. The latter allows you to constantly ask questions and solutions to improve the therapeutic act and/or to improve the quality of life of the human being. Insecurity forces the clinician to observe and reason not with predetermined patterns, avoiding the mistake of making the patient adapt to the clinician (it is always the clinician who must adapt to the patient). The future of osteopathy should also add notions deriving from quantum physics to the wealth of knowledge acquired during university studies. The macroscopic is influenced by the nanoscopic [5].
